# Effect of Substituting Soybean Meal in *Oreochromis niloticus* Diets with Pumpkin (*Cucurbita maxima*) Seed Cake on Water Quality, Growth, Antioxidant Capacity, Immunity, and Carcass Composition

**DOI:** 10.3390/ani14020195

**Published:** 2024-01-07

**Authors:** Hossam A. M. Mounes, Zeinab M. A. Abd-El Azeem, Dena. A. Abd El-Bary, Adham A. Al-Sagheer, Yasmina M. Abd-Elhakim, Bayan A. Hassan, Sherif S. Sadek, Kareem. M. Ahmed

**Affiliations:** 1 Limnology Department, Central Laboratory for Aquaculture Research, Agricultural Research Center, Abassa, Abu Hammad 44662, Egypt; 2 Fish Production Branch, Department of Animal Production, Faculty of Agriculture, Ain Shams University, Cairo 11241, Egypt; 3 Regional Center for Food and Feed, Agricultural Research Center, Giza 12619, Egypt; 4 Department of Animal Production, Faculty of Agriculture, Zagazig University, Zagazig 44511, Egypt; 5 Department of Forensic Medicine and Toxicology, Faculty of Veterinary Medicine, Zagazig University, Zagazig 44519, Egypt; yasmina.forensic@yahoo.com; 6 Pharmacology Department, Faculty of Pharmacy, Future University, Cairo 11835, Egypt; bayan.saffaf@fue.edu.eg; 7 Aquaculture Consultant Office, 9 Street No. 256, New-Maadi, Cairo 11435, Egypt; sadek_egypt35@hotmail.com

**Keywords:** pumpkin seed cake, soybean meal, Nile tilapia, growth, antioxidant

## Abstract

**Simple Summary:**

Soybean meal is a widely utilized protein source in aquatic feeds; however, its application in fish diets may be restricted due to elevated prices; market fluctuations; and heightened human, poultry, and livestock consumption. Therefore, utilizing unused agro-industrial by-products as alternative protein sources can reduce dependence on soybean meal, promote economic viability in tilapia farming, and address sustainability issues in aquaculture. This research examined the effects of substituting soybean meal with pumpkin seed cake in Nile tilapia diets concerning growth, water quality, antioxidant capacity, immune status, and body composition. The findings indicate that replacing 40% of dietary soybean meal protein with pumpkin seed cake can lead to significant improvements in growth, antioxidant capacity, and immune status in Nile tilapia fish.

**Abstract:**

A 10-week feeding experiment was performed to determine the impacts of partial substitution of soybean meal (SB) with pumpkin seed cake (PSC) in *Oreochromis niloticus* diets on water quality, growth rate, antioxidant capacity, immunity, and carcass composition. One hundred and fifty tilapia fish (average weight, 11.93 ± 0.17 g) were randomly allocated to five diets. The first diet (the basal diet) contained 420 g of SB per kg of feed. The remaining four diets, namely, D1, D2, D3, and D4, had SB partially replaced by PSC at 10%, 20%, 30%, and 40%, respectively. The results revealed that D4 and D1 significantly improved dissolved oxygen levels, while water temperature, pH, total ammonia, and nitrate levels were not significantly affected. Replacing SB with PSC significantly improved specific growth performance indicators and feed conversion compared to the control, with the D4 group showing the best values. Increasing PSC levels decreased serum glucose, aspartate aminotransferase, alanine aminotransferase, cholesterol, and triglyceride levels. In contrast, the D4 group had higher globulin, albumin, total protein, and lysozyme serum levels. Moreover, fish-fed PSC had significantly increased superoxide dismutase, glutathione peroxidase, and catalase activities and significantly decreased malondialdehyde levels. Increasing PSC substitution levels in fish diets increased the ash and crude lipid contents in the bodies of the fish, while crude protein and moisture decreased. In conclusion, replacing SB with PSC in fish diets significantly enhances growth performance, feed conversion, and fish health. Moreover, the findings suggest that PSC can be a promising alternative protein source for sustainable aquaculture practices.

## 1. Introduction

Aquaculture is an important industry that provides a significant source of protein for human consumption [1]. Nile tilapia (*Oreochromis niloticus*) is the world’s third-most commonly farmed fish species. It played a significant role, contributing approximately 5% to the total production of fish aquaculture and surpassing a cumulative output of 4.41 million tons in 2020 [2]. It is considered one of the world’s most extensively farmed fish species due to its rapid growth rate, adaptability to various environments, and high meat quality [3,4]. After China and Indonesia, Egypt is the third-largest producer of Nile tilapia globally [5]. However, the high cost of feed, especially protein sources such as soybean meal, is a major constraint on the sustainable development of tilapia farming [6]. Soybean meal (SM) is a beneficial source of protein in animal nutrition due to its superior amino acid profile and high crude protein content [7]. The increasing demand for SM and market price increases have prompted the aquaculture sector to search for environmentally friendly and cost-effective substitute feed ingredients [8,9,10]. Therefore, using unutilized agro-industrial by-products as alternative protein sources can reduce dependence on SM, ensure the economic viability of tilapia farming, and address sustainability challenges in aquaculture [6,11].

Pumpkin seed cake (PSC), produced when pumpkin seed oil is extracted, is rich in protein, fiber, and minerals [12]. Pumpkin seeds are pressed on a continuous screw press to produce PSC without affecting their nutrient contents [13]. Pumpkin seeds are known for various biological activities, including antioxidant, antimicrobial, anti-inflammatory, and antiparasitic effects [14]. In an earlier study, the nutritional composition of PSC was compared to that of SM [15]. The authors demonstrated that the gross energy, crude protein, and ether extract contents of PSC (59.8%, 12.46%, and 21.9 MJ/kg DM, respectively) were greater than those of SM (47.42%, 2.83%, and MJ/kg DM, respectively). Proteins isolated from PSC exhibited significantly higher concentrations of total sulfur amino acids than SM. Furthermore, PSC contains an abundant concentration of unsaturated fatty acids, including linoleic acid (50.9%), stearic acid (6.7%), palmitic acid (14.8%), and oleic acid (25.8%) [16,17]. Moreover, PSC has a low moisture content of about 7%, which makes it a suitable feed constituent for long-term storage and transport. In addition to being a source of protein, PSC is a prospective nutraceutical containing bioactive substances with beneficial health advantages, such as squalene, tocopherols, and phytosterols [12,16,18]. Utilizing by-products such as PSC in animal feeds can provide an economic benefit due to their reduced market price and provide feed producers with alternative feed ingredients. Nevertheless, limited studies have investigated the potential of PSC as a feed ingredient in practical fish feeds [17,19,20]. Furthermore, the results of these reports are inconsistent and require further investigation.

It has been hypothesized that replacing SB with PSC in tilapia diets would decrease SB usage without significantly impacting fish health or performance. The present study evaluated whether replacing SM with PSC would affect water quality, growth indicators, diet utilization, carcass composition, blood biochemical, immunity, or antioxidant status. Our research provides essential information on the prospective utilization of PSC as a sustainable protein alternative in tilapia feeds.

## 2. Materials and Methods

### 2.1. Diet Preparation

The PSC used in the present study consisted of 88.10% dry matter, 10.78% crude lipid, 55.20% crude protein, and 10.72% ash. PSC and the rest of the feed ingredients in this study were obtained from a local market in Sharkia Governorate, Egypt. Five iso-nitrogenous (crude protein, 315 g kg^−1^) and isolipidic (crude lipid, 65 g kg ^−1^) diets were formulated. The basal diet consisted of 420 g of SB per kg of diet, while in the remaining four diets, PSC replaced SB at 10%, 20%, 30%, and 40% (D1, D2, D3, and D4, respectively). All ingredients were ground into a fine powder, passed through a 200 µm mesh, and mixed thoroughly with vegetable and fish oils and water. The mixture was then pelletized with a feed mill (California Pellet Mill, San Francisco, CA, USA), dried for around a day in an oven until the dry matter levels reached about 90%, packed in sealed bags, and kept in a refrigerator at −20 °C until needed. All trial diets were prepared to meet the nutrient needs of tilapia, according to the recommendations of NRC [21]. Table 1 displays the nutrient composition and formulation of the diets.

### 2.2. Fish and Rearing Conditions

Before the experiment’s initiation, fiberglass tanks were cleaned and filled with dechlorinated tap water from the storage tank. Healthy Nile sex-reversed tilapia fingerlings were acquired from a commercial farm in Sharkia Governorate, Egypt. Fish were acclimated for ten days prior to the start of the experiment, during which they were fed the control diet three times a day, at 9:00, 13:30, and 17:00, until they reached apparent satiety. After acclimation, 150 juvenile (average weight, 11.93 ± 0.17 g) fish were randomly allocated to five groups, and each group had three fiberglass aquaria (water volume: 72 L; 10 fish per aquarium). The experimental diets (control, D1, D2, D3, and D4) were fed to the fish for 70 days, with the same schedule and procedures as during the acclimatization phase. The study was conducted with a photoperiod of 12 h light/12 h darkness. Every two days, 50% of the tank’s water was siphoned to eliminate suspended and dissolved wastes in the tanks, and a 0.5 Hp air blower continuously aerated the experimental tanks. Throughout the experiment, fish were hand-fed thrice daily (9:00, 13:30, and 17:00) at a feeding rate of 3% body weight to apparent satiation. Feed intake was adjusted per the new body mass every two weeks throughout the trial.

### 2.3. Water Quality Parameters

Every two weeks, physicochemical parameters were evaluated. At a depth of 20 cm, water samples from each aquarium were collected. pH was measured on-site using a digital pH meter (Fisher Scientific, Waltham, MA, USA). Dissolved oxygen and water temperature were determined using an oxygen and temperature meter (Jenway, London, UK). The ammonia concentration in the water samples was estimated by a multi-parameter analyzer (HANNA Instruments, Smithfield, RI, USA). According to APHA [22], the nitrate concentration was measured with a spectrophotometer (model Milton Roy 21D) at wavelengths of 410 nm.

### 2.4. Growth, Feed Utilization, and Body Indices

On the 70th day, fish were harvested, counted, and weighed in each aquarium. Livers and viscera were removed (3 fish/aquarium; *n* = 9 fish/treatment) and weighed to compute the viscerosomatic index (VSI) and hepatosomatic index (HSI). Growth and body indices such as feed conversion ratio (FCR), body weight gain (BWG), specific growth rate (SGR), VSI, and HSI were calculated using the following standard formula:

(1) $$\mathrm{BWG}\;(g\;{\mathrm{fish}}^{-1})=\frac{final\;wet\;weight-initial\;wet\;weight}{fish\;number\;per\;aquarium}$$

(2) $$\mathrm{SGR}\;(\%/\mathrm{day})=\frac{Ln\;of\;final\;wet\;weight-Ln\;of\;initial\;wet\;weight}{period(day)}\times 100$$

(3) $$\mathrm{FCR}=\frac{total\;feed\;intake(g)}{total\;BWG(g)}$$

(4) $$\mathrm{VSI}\;(\%)=\frac{wet\;weight\;of\;viscera}{fish\;wet\;weight}\times 100$$

(5) $$\mathrm{HSI}\;(\%)=\frac{wet\;weight\;of\;hepatopancreas}{fish\;wet\;weight}\times 100$$

### 2.5. Chemical Composition of the Diets and the Whole Carcass of Tilapia

Following the 10-week feeding experiment, three fish from each aquarium were sampled and frozen at −20 °C for chemical composition analysis of the carcass. The crude lipid, ash, moisture, and crude protein contents of the fish carcasses and experimental diets were estimated using AOAC [23] procedures. The moisture content was determined after three hours of dehydrating at 105 °C and a constant weight in an electric oven (JSON-100, Gongju, Republic of Korea). Five hours of combustion at 550 °C in a muffle furnace (Type 47900, Thermo Scientific, Waltham, MA, USA) were used to evaluate the ash content. The total lipid content was measured using a Soxhlet apparatus (El-Gomhouria Co., Zagazig, Egypt) and an ether extraction. After sulfuric acid digestion, the Kjeldal method was utilized to determine the crude protein level (N × 6.25).

### 2.6. Serum Biochemical, Immunity, and Antioxidant Parameters

At the end of the feeding trial, the fish were fasted for 24 h. Then, three fish per aquarium were anesthetized with MS-222 (100 mg/L) for blood sample collection from the caudal veins using non-heparinized syringes. The samples were centrifuged at 5000× *g* for 15 min at 4 °C to separate the serum, which was then stored at −20 °C for further examinations. The enzymatic activity of glutathione peroxidase (GPx), catalase (CAT), and superoxide dismutase (SOD), as well as the malondialdehyde (MDA) content, were measured by commercial kits from BioSource Inc., San Diego, CA, USA (Catalog number MBS480417, MBS2540413, MBS9718960, and MBS2540409, respectively). Lysozyme (LZM) activity was determined in line with the procedures of Ghareghanipoora et al. [24]. Serum total lipids, glucose, total protein (TP), albumin (ALB), creatinine, and uric acid concentrations were assessed colorimetrically using commercial kits (Bio-Diagnostics, Cairo, Egypt) with the catalog numbers TL 20 10, GL 13 20, TP 20 20, AB 10 10, CR 12 50, and UA 21 20, respectively. Globulin content was computed by subtracting ALB levels from TP levels. Serum alanine (ALT) and aspartate aminotransferase (AST) activities were quantified using Bio-diagnostics (Cairo, Egypt) colorimetric kits (AL 10 31 (45) and AS 10 61 (45), respectively).

### 2.7. Statistical Analysis

The significance of growth, feed utilization, body indices, water quality, carcass composition, and blood contents was discovered via one-way ANOVA using SAS software (Version 9.00) [25]. Then, Tukey’s post hoc test was used. The optimal level of substitution using PSC was determined through the use of polynomial regression analysis, as explained by Yossa and Verdegem [26]. The third-order polynomial regression (cubic) provided a better fit to the observed patterns in our dataset compared to linear or quadratic models. Statistical significance was set at *p <* 0.05. In addition, correlations among growth performance indicators, the carcass composition, and serum biochemical parameters were examined by the Pearson coefficient of pairwise comparison between samples. They were recognized from the correlation matrix heatmap produced via GraphPad Prism version 8 (GraphPad Software, San Diego, CA, USA) [27].

## 3. Results

### 3.1. Water Quality

**Figure 1:**
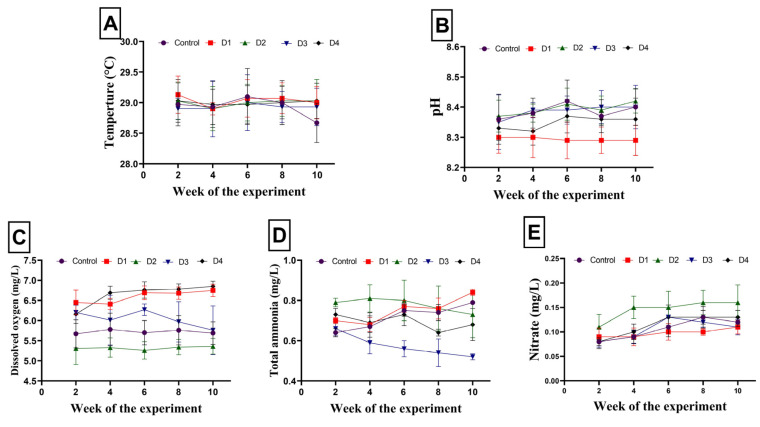
Average values of water quality parameters, determined every second week during the trial, including: (**A**) temperature, (**B**) pH, (**C**) dissolved oxygen, (**D**) total ammonia, and (**E**) nitrate. Control, D1, D2, D3, and D4 indicate that 0%, 10%, 20%, 30%, and 40% of soybean meal were replaced by pumpkin seed cake (PSC), respectively. Values are expressed as means ± standard error.

Figure 1A–E displays the estimated water quality parameters determined every second week throughout the experimental period. The values of temperature, pH, dissolved oxygen, ammonia, and nitrate were in the ranges of 28.67–29.13 °C, 8.29–8.42, 5.26–6.85 mg/L, 0.52–0.84 mg/L, and 0.08–0.16 mg/L, respectively, during the trial.

### 3.2. Growth Performance and Body Indices

**Figure 2:**
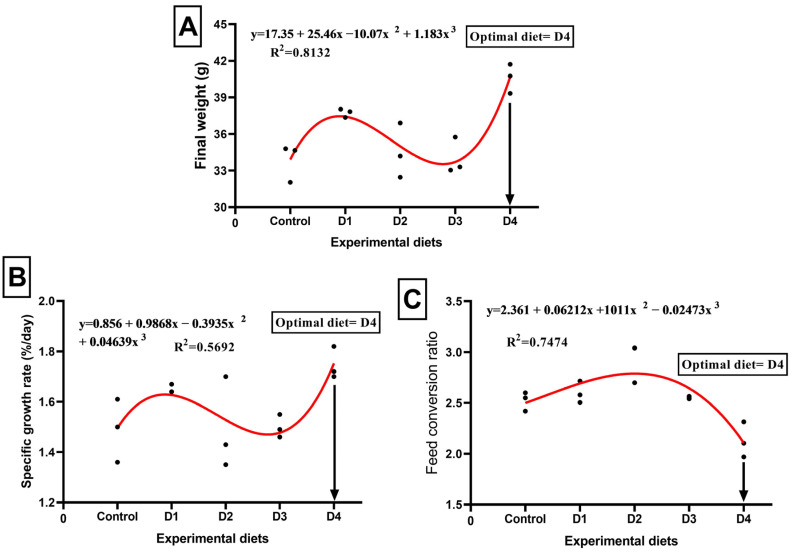
Relationship between final fish weight (**A**), specific growth rate (**B**), and feed conversion ratio (**C**) of Nile tilapia (*Oreochromis niloticus*) and different levels of pumpkin seed cake (PSC). Control, D1, D2, D3, and D4 indicate that 0%, 10%, 20%, 30%, and 40% of SM were replaced by PSC, respectively.

**Figure 3:**
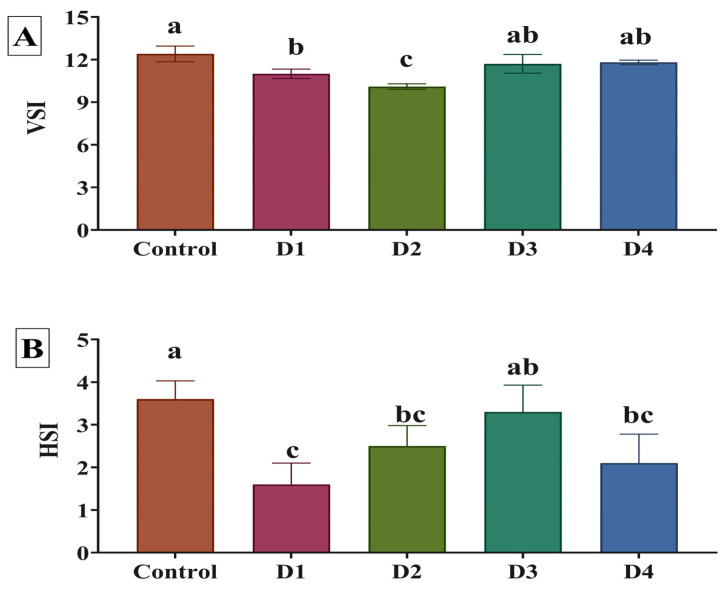
Viscerosomatic index (VSI; **A**) and hepatosomatic index (HSI; **B**) as affected by substituting soybean meal (SM) with pumpkin seed cake (PSC) in Nile tilapia diets. Control, D1, D2, D3, and D4 indicate that 0%, 10%, 20%, 30%, and 40% of SM were replaced by PSC, respectively. ^a–c^ Means in bars with different superscripts differ significantly. Different letters above the error bar indicate significant differences at *p*  <  0.05, as determined by one-way ANOVA followed by Tukey’s multiple comparisons test. Values are expressed as means ± standard deviation (3 fish/aquarium; *n* = 9 fish/treatment).

Table 2 illustrates the growth indicators of *O. niloticus* fed the different experimental diets after 70 days of the experiment. The growth and feed efficiency parameters of Nile tilapia were enhanced by PSC-based diets compared to the control diet. The D4 group exhibited the greatest improvements in final weight, BWG, SGR, and FCR when PSC was substituted for SB. Feed intake was significantly (*p* < 0.05) increased in the fish fed on the D1 and D2 diets. The relationships between SGR, final weight, and FCR, as well as the different substitution levels using PSC, were best expressed by third-order polynomial regression (cubic) equations, where final weights were as follows: y = 17.35 + 25.46x − 10.07x^2^ + 1.183x^3^, R^2^ = 0.8132 (Figure 2A); SGR: y = 0.856 + 0.9868x −0.3935x^2^ + 0.04639x^3^, R^2^ = 0.5692 (Figure 2B); and FCR: y = 2.361 + 0.06212x + 1011x^2^ − 0.02473x^3^, R^2^ = 0.7474 (Figure 2C). Based on the polynomial regression analysis, the optimal substitution level using PSC for the maximum final weight and SGR and the best FCR were found in the fish fed on the D4 diet. As displayed in Figure 3, VSI and HSI showed significant differences in favor of PSC treatments compared to the control treatment. The lowest VSI value was detected in the D2 group, while the highest was found in the control treatment group. The highest HSI record was found in the control treatment, while the lowest was recorded in the D1 group.

### 3.3. Carcass Composition

Table 3 displays the whole-body composition of *O. niloticus* fed the experimental diets which substituted SB with PSC for 70 days. Increasing PSC substitution levels in fish diets increased the ash (except the D4 group) and crude lipid contents in the bodies of the fish. In contrast, the crude protein and moisture contents in the fish decreased as PSC substitution increased in their diets.

### 3.4. Blood Biochemical Biomarkers

The results concerning blood biochemical biomarkers, as affected by SB’s replacement with PSC, are presented in Table 4. Reduced total cholesterol and triglyceride concentrations were observed as a dose–response with increasing PSC levels in the diets, and the D4 group had the lowest values (*p* < 0.05). Following the same trend, the serum contents of ALT, AST, creatinine, and urea were significantly decreased with all dietary inclusion levels of PSC. Significantly (*p* < 0.05) lower glucose was detected in the D4-fed fish compared to the control group. Total protein and globulin levels in the blood of fish fed the D1 and D4 diets were significantly (*p* < 0.05) higher than those of fish fed other experimental diets. In contrast, albumin levels in the blood of fish fed the D1, D3, and D4 diets were significantly (*p* < 0.05) higher than those of fish fed the control diet (Table 4).

### 3.5. Antioxidant Activity and Immune Status

**Figure 4:**
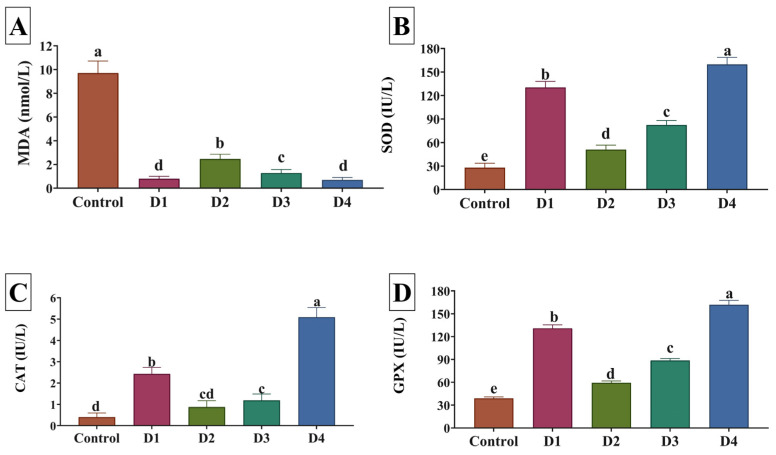
The serum levels of (**A**) malondialdehyde (MDA), (**B**) superoxide dismutase (SOD), (**C**) catalase (CAT), and (**D**) glutathione peroxidase (GPx) as affected by substituting soybean meal (SBM) with pumpkin seed cake (PSC) in Nile tilapia diets. Control, D1, D2, D3, and D4 indicate that 0%, 10%, 20%, 30%, and 40% of SM were replaced by PSC, respectively. Different letters above the error bar indicate significant differences at *p*  <  0.05, as determined by one-way ANOVA followed by Tukey’s multiple comparisons test. Values are expressed as means ± standard deviation (*n* = 9/treatment).

**Figure 5:**
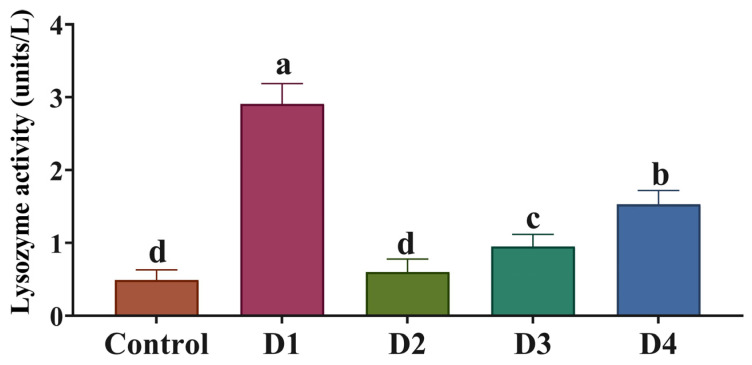
Lysozyme activity as affected by substituting soybean meal (SBM) with pumpkin seed cake (PSC) in Nile tilapia diets. Control, D1, D2, D3, and D4 indicate that 0%, 10%, 20%, 30%, and 40% of SM were replaced by PSC, respectively. Different letters above the error bar indicate significant differences at *p*  <  0.05, as determined by one-way ANOVA followed by Tukey’s multiple comparisons test. Values are expressed as means ± standard deviation (*n* = 9/treatment).

SOD, GPx, and CAT activities were markedly higher (*p* < 0.05) in all dietary incorporation levels of PSC relative to the control group (Figure 4). Notably, their highest values were detected in the D4 group. Conversely, reduced MDA levels were detected with all PSC substitution levels in diets, and the D1 and D4 groups had the lowest values (*p* < 0.05). Regarding lysozyme activity, fish fed PSC diets exhibited significantly (*p* < 0.05) higher activity than those fed the control diet (Figure 4). Fish fed the D1 diet had the most increased lysozyme activity in their serum compared with those fed the other diets (Figure 5).

### 3.6. Pearson Correlation between the Estimated Parameters

**Figure 6:**
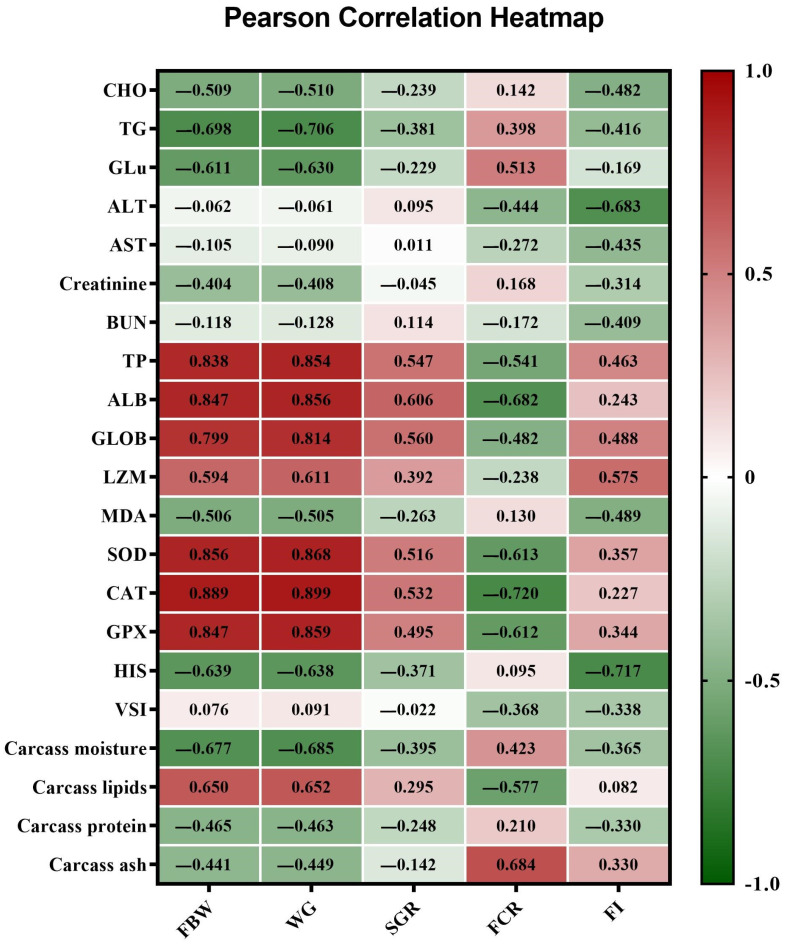
The correlation matrix heatmap shows the values of the Pearson correlation coefficient among growth performance indicators, carcass composition, and serum biochemical parameters. The positive values are in red, and the negative values are in green. The values range from −1 to 1, whereby 1 indicates a perfect negative linear relationship between variables, 1 indicates a perfect positive linear relationship between variables, and 0 indicates no relationship between studied variables. FBW: final body weight; WG: weight gain; SGR: specific growth rate; FCR: feed conversion ratio; and FI: feed intake; CHO: cholesterol; TG: triglycerides; GLU: glucose; ALT: alanine transaminase; AST: aspartate aminotransferase; BUN: blood urea nitrogen; TP: total protein; ALB: albumin; GLOB: globulin; LZM: lysozyme; MDA: malondialdehyde; SOD: superoxide dismutase; CAT: catalase; GPx: glutathione peroxidase, HIS: hepatosomatic index; and VSI: viscerosomatic index.

As revealed in the correlation matrix heatmap in Figure 6, a significant (*p* < 0.01) positive correlation was detected between the growth performance indices, including FBW and WG, and the serum levels of antioxidant enzymes (SOD, CAT, and GPx), proteins (TP, ALB, and GLOB), and the lysozyme activity. On the contrary, FBW and WG were negatively correlated with TG, GLU, HSI, and carcass moisture content. Furthermore, FCR was negatively correlated with SOD, CAT, GPx, and ALB.

## 4. Discussion

Our findings showed that all water quality parameters, including water temperature, pH, total ammonia, dissolved oxygen, and nitrate, as estimated in all experimental groups, were within the recommended limits for warm-water fish [28,29].

Recent research has focused on the viability of incorporating agro-industrial and plant-based feed ingredients into fish feeds to gain financial advantages while enhancing the sustainability of aquaculture [30]. Several research investigations have found that replacing SB with non-traditional feed constituents had no detrimental impact on the growth of rainbow trout [31,32], grass carp [33,34], Nile tilapia [6], or common carp [35]. In the current experiment, fish fed the D4 diet showed better FW, WG, SGR, and FCR. Recently, Musthafa et al. [36] stated that the *Mucuna pruriens* seed meal substantially enhanced the immune status and growth of the freshwater fish *O. niloticus*. Furthermore, Bai et al. [37] found that a diet containing 30% cottonseed meal improved the growth indicators of the red-bellied Pacu. The enhanced growth observed in fish fed with PSC could be attributed to this alternative feed ingredient’s nutritional and bioactive properties. PSC is rich in protein, essential amino acids, and minerals [12], all of which are essential nutrients for the growth and development of fish. In addition, PSC contains bioactive compounds such as phytosterols and phenolic compounds [38] that have been shown to promote fish health and growth. Another important perspective is that the feed intake has significantly improved in most fish groups fed PSC-containing diets, which could be responsible for the recorded growth. Additionally, Greiling et al. [20] have demonstrated that increased intake of the PSC-containing diet by African catfish could be related to increased feed palatability.

VSI and HSI showed significant differences in favor of PSC inclusion compared to the control. Our findings contradicted those of Sezgin and Aydın [17], who concluded that PSC had no significant influence on HSI or VSI. However, our result could be explained by several studies, including those of Caili et al. [39], Nkosi et al. [40], Bardaa et al. [16], Musthafa et al. [41], and Salehi et al. [14], which suggest that the observed improvements in VSI and HSI in tilapia fed with diets containing PSC are likely due to a complex interplay of metabolic processes influenced by the nutritional composition of the feed. As a result, this could positively impact various metabolic processes, including those involved in growth.

Fish carcass composition can be influenced by changes in the composition of their diets [4]. In the present study, increasing PSC substitution levels in fish diets increased the ash and crude lipid contents, but reduced the crude protein and moisture contents. Likewise, substitution with other plant protein sources resulted in a comparable change in the carcass composition of Nile tilapia [4,6]. The increase in crude lipid content could be related to the increased feed intake, which has been shown to increase whole-body energy retention in the form of increased whole-body lipid deposition. In this regard, Greiling et al. [20] reported that the higher energy content of the PSC-containing diets could increase the weights of different sites of lipid deposition in fish carcasses. Meanwhile, the reduction in protein content could be related to the presence of some anti-nutritional factors or the deficiency of some amino acids, such as lysine [15].

Blood biochemical parameters are a conventional technique used regularly to evaluate the physiological, pathological, and immunological statuses of fish [42]. Herein, glucose, total cholesterol, and triglyceride concentrations were significantly reduced in *O. niloticus* fed with PSC diets, and the D4 group had the lowest values. The results of this study corroborate those of Caili et al. [39], who found that pumpkin seeds could efficiently reduce serum triglyceride levels and cholesterol. Similarly, Sezgin and Aydın [17] found that increasing the inclusion level of PSC in mirror carp (*Cyprinus carpio*) diets led to a decrease in both cholesterol and triglyceride levels, with a continued effect on cholesterol until reaching 100% inclusion. This notable reduction could be attributed to PSC’s phytosterol bioactive components, which might affect both the expression levels of genes related to cholesterol and lipid metabolism and the activity of hepatic enzymes [17,43]. Our observation on serum glucose levels was similar to the findings of Sezgin and Aydın [17], who noted a decrease in serum glucose levels in groups fed on diets containing 66% and 100% PSC. In addition, the serum contents of ALT, AST, creatinine, and urea were significantly decreased with all dietary inclusion levels of PSC. The antioxidant activity of PSC may be responsible for its hepatoprotective and nephroprotective effects [44]. Major functions of serum proteins include sustaining pH, osmotic pressure, and transporting metabolites. TP is crucial to the innate immune response and humoral immunity of fish [45]. In this study, the TP and globulin levels in the blood of fish fed the D1 and D4 diets were significantly higher than in those fed other experimental diets. In contrast, the ALB levels of fish fed the D1, D3, and D4 diets were significantly higher than those fed the control diet. Our dataset is in contrast with the findings of Sezgin and Aydın [17], who found that the serum TP, ALB, ALT, and AST levels underwent only numerical changes in those parameters’ values, with no statistically significant effect of PSC inclusion in the diets of mirror carp.

The antioxidant activity of enzymes, such as GPx, CAT, and SOD, is an essential biomarker for monitoring fish health and reactivity to external stimuli [30], and it can be used to assess fish antioxidant capability. Furthermore, Al-Sagheer et al. [46] stated that MDA, a marker of lipid peroxidation, can be utilized to assess the level of oxidative stress and antioxidant status. In this investigation, the activity of SOD, CAT, and GPx significantly increased while MDA decreased in fish fed formulated feeds with PSC. The addition of PSC, which contains active ingredients like proteins, minerals, phytosterols, triterpenes, carotenoids, lignans, polyunsaturated fatty acids, tocopherol, and phenolic and antioxidative compounds, may have triggered antioxidant defense and decreased MDA lipid oxidation in all treated groups [14,16,39,40]. Previous studies have found that PSC has potent antioxidative properties, as shown by its free radical scavenging activity and augmentation of antioxidase activity [47,48]. Furthermore, these effects could be attributed to the essential fatty acids found in pumpkin seeds, which play a critical role in maintaining cell membrane integrity and regulating gene expression, as well as the antioxidants found in pumpkin seeds, which could help to protect fish from oxidative stress by scavenging free radicals and reducing inflammation [20].

Lysozyme is a positively charged protein found in various bodily fluids, such as mucus, lymphoid tissue, and plasma, and is synthesized in multiple fish tissues [49]. It is essential to multiple defense mechanisms, including the immune response, opsonization, bacteriolysis, and antimicrobial activity [36]. Our dataset showed that lysozyme activity was boosted by incorporating PSC into feed compared to control groups. The findings of Musthafa et al. [41] on *O. mossambicus* fed diets including *C. mixta* seed meal corroborate these findings, since they showed an enhanced immune response to *A. hydrophila* and the Gram-negative bacterium. There is a strong link between oxidative stress and immune function in fish [50], and the liver plays an important role in synthesizing immune proteins [51]. The improved hepatic function and the antioxidant capacity of fish fed diets containing PSC in the current experiment could also have partly contributed to the recorded enhancement of the immune status. Notably, the positive relationship detected by the Pearson correlation analysis between growth indices and the antioxidant enzymes and lysozyme activity indicates that the enhancement of these parameters could be collectively responsible for the recorded enhanced growth performance and nutrient utilization of fish fed diets containing PSC.

## 5. Conclusions

Based on the study’s findings, partial SB substitution (particularly 40% replacement) with PSC in the diets of Nile tilapia significantly enhanced growth performance, feed conversion, water quality, antioxidant capacity, and immunity. This study concludes that PSC could be a promising, sustainable, and cost-effective alternative protein source for Nile tilapia diets. Additional research is required in order to investigate the long-term effects of PSC on fish health and the environment.

## Figures and Tables

**Table 1:** Ingredients and proximate composition of the experimental diets (g kg^−1^) on a dry matter basis.

	Experimental Diets ^1^				
	Control	D1	D2	D3	D4
Ingredients					
Soybean meal	420	378	336	294	252
Pumpkin seed cake ^2^	0	33.5	67	100.4	133.9
Yellow corn	375	375	375	375	375
Fish meal	100	100	100	100	100
Wheat bran	70	70	70	70	70
Cellulose	0	11.2	22.4	33.7	44.9
Fish oil	10	10	10	10	10
Sunflower oil	20	17.3	14.6	11.9	9.2
Premix ^3^	5	5	5	5	5
Proximate composition					
Dry matter	914.0	915.0	912.0	921.0	925.0
Crude protein (N × 6.25)	315.0	316.5	314.5	315.5	314.5
Crude lipid	65.5	65.7	65.4	65.1	65.9
Ash	75.5	79.5	76.5	74.5	76.0

^1^ Control, D1, D2, D3, and D4 indicate that 0%, 10%, 20%, 30%, and 40% of soybean meal were replaced by pumpkin seed cake, respectively. ^2^ Pumpkin seed cake: 55.20% crude protein, 10.78% crude lipid, and 10.72% ash. ^3^ Provides the following per kg of diet: vitamin A 4000 IU, vitamin D_3_ 600 IU, vitamin E 20 mg, vitamin K_3_ 5 mg, vitamin B_1_ 3.6 mg, vitamin B_2_ 6 mg, vitamin B_5_ 12 mg, vitamin B_6_ 3.5 mg, vitamin B_12_ 0.02 mg, vitamin B_3_ 14.4 mg, biotin 0.07 mg, folic acid 0.9 mg, inositol 300 mg, vitamin C 50 mg, Mg 15 mg, Fe 30 mg, Zn 42 mg, Cu 4 mg, K 75 mg, Co 0.11 mg, Mn 1.6 mg, Se 0.04 mg, Mo 0.005 mg, and I 0.4 mg.

**Table 2:** Growth performance and nutrient utilization as affected by substituting soybean meal (SBM) with pumpkin seed cake (PSC) in Nile tilapia diets.

Items	Experimental Diets ^1^					SDM ^2^
	Control	D1	D2	D3	D4	
Initial weight (g fish^−1^)	11.86 ^NS^	11.87	12.08	11.92	11.94	0.17
Final weight (g fish^−1^)	33.83 ^c^	37.74 ^b^	34.51 ^c^	34.02 ^c^	40.60 ^a^	3.01
Body weight gain (g fish^−1^)	21.97 ^c^	25.87 ^b^	22.43 ^c^	22.10 ^c^	28.66 ^a^	2.98
Specific growth rate (%)	1.51 ^b^	1.65 ^ab^	1.51 ^b^	1.51 ^b^	1.75 ^a^	0.20
Feed intake (g fish^−1^)	55.36 ^b^	67.26 ^a^	65.40 ^a^	56.41 ^b^	61.90 ^ab^	5.38
Feed conversion ratio (g feed/g gain)	2.52 ^ab^	2.60 ^ab^	2.93 ^a^	2.55 ^ab^	2.16 ^b^	0.29
Survival rate (%)	100 ^NS^	100	100	100	100	0.00

^1^ Control, D1, D2, D3, and D4 indicate that 0%, 10%, 20%, 30%, and 40% of SM were replaced by PSC, respectively. ^2^ SDM stands for the standard deviation of the mean. ^a–c^ Means in rows with different superscripts differ significantly (*p* < 0.05). NS = no significant differences between groups (*p* > 0.05).

**Table 3:** Carcass composition as affected by substituting soybean meal (SBM) with pumpkin seed cake (PSC) in Nile tilapia diets (% on a dry matter basis, except for moisture on a wet basis).

Items	Experimental Diets ^1^					SDM ^2^
	Control	D1	D2	D3	D4	
Moisture	76.9 ^a^	72.4 ^cd^	74.9 ^b^	73.0 ^c^	71.8 ^d^	1.9
Crude lipids	14.4 ^d^	18.0 ^c^	16.8 ^c^	20.3 ^b^	23.7 ^a^	3.4
Crude protein	64.8 ^a^	58.7 ^c^	60.0 ^b^	57.4 ^d^	57.5 ^d^	2.8
Ash	20.0 ^b^	22.5 ^a^	22.5 ^a^	21.8 ^a^	18.3 ^c^	1.9

^1^ Control, D1, D2, D3, and D4 indicate that 0%, 10%, 20%, 30%, and 40% of SM were replaced by PSC, respectively. ^2^ SDM stands for the standard deviation of the mean. ^a–d^ Means in rows with different superscripts differ significantly (*p* < 0.05).

**Table 4:** Blood biochemical parameters, as affected by substituting soybean meal (SBM) with pumpkin seed cake (PSC) in Nile tilapia diets.

Items	Experimental Diets ^1^					SDM ^2^
	Control	D1	D2	D3	D4	
Total cholesterol (mg/dL)	178.0 ^a^	126.25 ^c^	139.25 ^b^	130.0 ^bc^	123.05 ^c^	6.39
Triglycerides (mg/dL)	280.81 ^a^	188.25 ^d^	235.0 ^b^	208.92 ^c^	173.25 ^d^	6.37
Glucose (mg/dL)	122.83 ^a^	112.05 ^ab^	122.25 ^a^	116.05 ^ab^	105.36 ^b^	7.48
ALT (U/L)	73.12 ^a^	62.18 ^c^	65.48 ^c^	67.01 ^bc^	69.09 ^b^	3.24
AST (U/L)	111.25 ^a^	87.5 ^b^	76.25 ^c^	78.5 ^c^	85.91 ^b^	3.78
Creatinine (mg/dL)	0.60 ^a^	0.40 ^bc^	0.47 ^b^	0.41 ^bc^	0.33 ^c^	0.23
Urea (mg/dL)	16.2 ^a^	10.15 ^b^	12.1 ^b^	11.75 ^bc^	12.0 ^b^	4.75
Total protein (g/dL)	1.38 ^b^	3.29 ^a^	1.33 ^b^	1.54 ^b^	3.09 ^a^	0.31
Albumin (g/dL)	0.80 ^c^	1.35 ^ab^	0.84 ^c^	1.08 ^b^	1.54 ^a^	0.28
Globulin (g/dL)	0.58 ^b^	1.94 ^a^	0.49 ^b^	0.46 ^b^	1.55 ^a^	0.35

^1^ Control, D1, D2, D3, and D4 indicate that 0%, 10%, 20%, 30%, and 40% of SM were replaced by PSC, respectively. ^2^ SDM stands for the standard deviation of the mean. ^a–d^ Means in rows with different superscripts differ significantly (*p* < 0.05). Alanine aminotransferase (ALT), aspartate aminotransferase (AST).

## Data Availability

The datasets used along with this research are available from the corresponding author upon reasonable request.
